# Seminological, Hormonal and Ultrasonographic Features of Male Factor Infertility Due to Genetic Causes: Results from a Large Monocentric Retrospective Study

**DOI:** 10.3390/jcm13154399

**Published:** 2024-07-27

**Authors:** Rossella Mazzilli, Simona Petrucci, Virginia Zamponi, Bianca Golisano, Giulia Pecora, Camilla Mancini, Gerardo Salerno, Laura Alesi, Ilaria De Santis, Fabio Libi, Carla Rossi, Marina Borro, Salvatore Raffa, Vincenzo Visco, Giuseppe Defeudis, Maria Piane, Antongiulio Faggiano

**Affiliations:** 1 Endocrinology and Andrology Unit, Department of Clinical and Molecular Medicine, Sapienza University of Rome, Sant’Andrea Hospital, 00189 Rome, Italy; rossella.mazzilli@uniroma1.it (R.M.); virginia.zamponi@uniroma1.it (V.Z.); bianca.golisano@uniroma1.it (B.G.); g.pecora@uniroma1.it (G.P.); endocrinologiastudycoordinator@gmail.com (C.M.); antongiulio.faggiano@uniroma1.it (A.F.); 2 Department of Molecular Medicine, Sapienza University of Rome, 00185 Rome, Italy; 3 Department of Clinical and Molecular Medicine, Sapienza University of Rome, Sant’Andrea Hospital, 00189 Rome, Italy; gerardo.salerno@uniroma1.it (G.S.); marina.borro@uniroma1.it (M.B.); salvatore.raffa@uniroma1.it (S.R.); vincenzo.visco1@uniroma1.it (V.V.); maria.piane@uniroma1.it (M.P.); 4 UOD Medical Genetics and Advanced Cell Diagnostics, Sant’Andrea Hospital, 00189 Rome, Italy; lalesi@ospedalesantandrea.it (L.A.); idesantis@ospedalesantandrea.it (I.D.S.); flibi@ospedalesantandrea.it (F.L.); crossi@ospedalesantandrea.it (C.R.); 5 Department of Theoretical and Applied Sciences, eCampus University, 22060 Novedrate, Italy; 6 Unit of Endocrinology and Diabetes, Department of Medicine, University Campus Bio-Medico di Roma, 00128 Rome, Italy

**Keywords:** male infertility, genetic, congenital hypogonadotropic hypogonadism, karyotype, Y chromosome microdeletion, *CFTR* gene

## Abstract

**Objectives:** Evaluate the prevalence of genetic factors in a large population of infertile subjects and define the seminological, hormonal, and ultrasonographic features for each alteration. **Methods**: This single-center retrospective study included male partners of infertile couples undergoing genetic investigations due to oligozoospermia or azoospermia evaluated from January 2012 to January 2022. The genetic investigations consist of karyotype, *CFTR* gene mutations plus variant of the IVS8-5T polymorphic trait, Y chromosome microdeletion, and Next Generation Sequencing panel to analyze genes implicated in congenital hypogonadotropic hypogonadism (CHH). **Results**: Overall, 15.4% (72/466) of patients received a diagnosis of genetic cause of infertility. Specifically, 23 patients (31.9%) harbor mutations in the *CFTR* gene, 22 (30.6%) have a 47, XXY karyotype, 14 (19.4%) patients show a Y chromosome microdeletion, 7 (9.7%) have structural chromosomal anomalies, and 6 (8.3%) have CHH. Overall, 80.6% of patients were azoospermic and 19.4% oligozoospermic (sperm concentration 3.5 ± 3.8 million/mL). Almost all patients presented hormonal alterations related to the specific genotype, while the main ultrasound alterations were testicular hypoplasia, calcifications/microcalcifications, and enlarged/hyperechoic epididymis. **Conclusions**: The prevalence of genetic abnormalities in males of infertile couples was 15.4% in our Center. *CFTR* gene disease-causing variants resulted in more frequent, with various clinical features, highlighting the complexity and heterogeneity of the presentation. Other investigations are needed to understand if conditions like ring chromosomes and other translocations are related to infertility or are incidental factors.

## 1. Introduction

Infertility is a disease of the male or female reproductive system defined by the failure to achieve a pregnancy after at least 12 months of regular unprotected sexual intercourse [1]. Despite a thorough diagnostic approach, the cause of infertility remains unknown in a large percentage of male partners of infertile couples. In this regard, along with several conditions, including a high sperm DNA fragmentation [2,3], an unknown genetic factor is speculated to be accountable for these unresolved cases. Overall, a known genetic cause is responsible for 15% of cases of male infertility [1,4,5,6,7,8]. These genetic alterations can lead to qualitative (sperm with low motility and/or with malformed morphology) or quantitative alterations of semen, causing oligozoospermia or azoospermia, both obstructive (OA) and non-obstructive (NOA) [6].

The diagnostic approach and genetic screening are different from case to case. In particular, males with congenital hypogonadotropic hypogonadism (CHH), a rare genetic disease caused by GnRH deficiency and characterized by absent or incomplete puberty with infertility, should be tested for pathogenic/likely pathogenic (P/LP) variants or exonic/multiexonic rearrangements of genes linked to the most frequent cause of Kallmann Syndrome [9]. Meanwhile, karyotype and Y chromosome microdeletions studies are indicated in patients with primary hypogonadism. Indeed, a chromosomal aberration, mainly a 47, XXY karyotype related to Klinefelter syndrome (KS), is found in about 5% of men with oligozoospermia and 10–15% of azoospermic men, while Y microdeletions are found in 3–7% of men with oligozoospermia and up to 15% of men with azoospermia [10].

Furthermore, testing for the most common mutations of the *cystic fibrosis transmembrane conductance regulator* (*CFTR*) gene should be performed in patients with congenital bilateral absence of the vas deferens (CBAVD) or in the presence of the suspicious of semen impairment due to obstructive cause [7].

Understanding and identifying the genetic cause is highly relevant for the correct evaluation of infertility and its appropriate management, including positive sperm retrieval prediction in OA and NOA [11,12], and it is crucial to estimate the risk of transmitting the defective gene through natural conception or assisted reproductive technique (ART) [13].

This work aims to estimate the prevalence of genetic factors and each genetic condition in a large population of male partners of infertile couples and to define for each disease the clinical, seminological, hormonal, and ultrasonographic features.

## 2. Materials and Methods

### 2.1. Patients

This retrospective observational monocentric study included patients who were consecutively referred to the Andrology Unit (Sant’Andrea Hospital, “Sapienza” University of Rome) for couple infertility and azoospermia or oligozoospermia (with a sperm concentration <10 Concentration ×10^6^/mL), from January 2012 to January 2022. Patients with normozoospermia and diagnosis of genetic alteration diagnosed only for hypogonadism or sexual dysfunction were excluded.

The study adhered to the Hospital’s Ethics Committee guidelines and the Ethical Principles for Medical Research Involving Human Subjects as adopted at the 18th WMA General Assembly, Helsinki, Finland, June 1964, and amended by the 55th WMA General Assembly, Tokyo, Japan, October 2004 and subsequent modifications when enforced (last, Fortaleza, Brazil, October 2013). Patients signed an informed consent form before the genetic test, expressing consent to use this information anonymously for research purposes (according to DL196/03 n.196).

### 2.2. Semen Analysis

The seminal fluid of each participant was collected by masturbation after sexual abstinence between 2 and 7 days. Semen analysis was carried out according to 2010 WHO guidelines, evaluating spermatozoa morphology, motility, and concentration [14]. The sample container was placed in an incubator (37 °C) for 30–60 min. The physical and chemical characteristics of the seminal liquid were then evaluated (appearance, pH, liquefaction, and viscosity).

### 2.3. A Genetic Testing

Genetic exams were conducted at the Medical Genetics Unit of Sant’Andrea Hospital. Genetic testing was performed in patients with azoospermia and severe oligozoospermia. Karyotype analysis, Y chromosome deletions, and hypogonadotropic hypogonadism were performed following guidelines indications [7]. *CFTR* gene mutations were researched in patients with OA from the perspective of ART treatment.

#### 2.3.1. Karyotype Analysis

Karyotype analysis was performed on peripheral blood lymphocyte cultures incubated at 37 °C in RPMI 1640 medium containing fetal bovine serum, phytohaemagglutinin, and penicillin-streptomycin for 72 h. After chromosomal harvesting by cell hypotonic shock, microscope slides were prepared. The cytogenetic analysis was performed on metaphase chromosomes using Giemsa-Trypsin-Giemsa (GTG) banding, with a resolution of 450–550 bands for each patient. At least 20 metaphases were evaluated for each patient using a computerized Karyotyper (Cytovision 2.7). Karyotypes were reported using the ISCN (International System for Human Cytogenetic Nomenclature) nomenclature.

#### 2.3.2. Y Chromosome Deletions

Genomic DNA was extracted from the peripheral blood samples using the DNeasy Blood & TissueKit and QIAamp DNA Blood Mini Kit (Hilden, Qiagen, Germany), according to the manufacturer’s instructions. The Y microdeletion was investigated using the polymerase chain reaction (PCR) technique. The amplifications of the AZFa, AZFb, and AZFc regions were obtained using 20 pairs of primers amplifying the nonpolymorphic short segments of DNA (sequence-tagged sites, STSs), combined in 5 multiplex PCR (SY14, SY181, SY84, SY86, SY121, SYPR3, SY124, SY127, SY128, SY130, SY133, SY134, SY145, SY152, SY157, SY242, SY208, SY254, SY 255, and SY157). The presence or absence of the 20 target sequences was assessed by gel electrophoresis in 4% NuSieve 3:1 agarose.

#### 2.3.3. *CFTR* Gene Mutations

Molecular analysis of the most frequent mutations of *CFTR* and the IVS8-5T polymorphic tract was performed by Inno-Lipa *CFTR* 19, *CFTR*17+Tn Update, and *CFTR* Italian Regional Kits (Innogenetics, Ghent, Belgium), providing the screening for 57 *CFTR* gene variants (from 2012 to 2017) and by Inno-Lipa CFTRiage (Innogenetics, Ghent, Belgium), that perform the analysis 88 *CFTR* variants (from 2018 to 2021), following the manufactures instructions. The identified variants were classified according to ACMG guidelines [11] and specific disease-related databases (http://www.cftr2.org accessed on 14 June 2024). All identified disease-causing variants were confirmed by repeating the testing.

#### 2.3.4. Congenital Hypogonadotropic Hypogonadism 

A target next-generation sequencing (NGS) panel was designed to analyze genes implicated in CHH development. A DNA library was prepared using the SureSelect Custom Constitutional Panel 17Mb Library Prep Kit (Agilent, Zaventem, Belgium) and sequenced with the Nextseq500/550 mud output V2 kit (Illumina, Antwerpen, Belgium) on theNextSeq500 platform (Illumina, Antwerpen, Belgium). 

The alignment of the sequences to the hg19 reference genome was performed with BWA and GATK (ver 4) tools. Variants were interpreted using the ANNOVAR and Alamut Software Suit (Interactive Biosoftware) programs. All the detected variants were classified according to ACGM criteria [15] and confirmed by Sanger sequencing.

### 2.4. Hormonal Profile

Blood samples were obtained at 8:00 a.m.; the plasma levels of luteinizing hormone (LH), follicle-stimulating hormone (FSH), thyroid-stimulating hormone (TSH), testosterone, estradiol, prolactin, and HbA1c were measured. Chemiluminescence microparticle immunoassay (CMIA) and immunoassay (CLIA) were used.

### 2.5. Testicular Ultrasound

A testicular ultrasound was performed in the Andrology Unit. The investigation was conducted with a 7 MHz probe with a Color-Doppler module. Testicular volume (the lower limit was 12 mL), echotexture (specifically, testicular inhomogeneity was defined as the presence of small hypoechoic foci and/or thin hypoechoic striae until diffuse inhomogeneity with “netting”/“geographical map” appearance), epididymal abnormalities (enlarged epididymis/hyperechogenicity), and varicocele were evaluated [16].

### 2.6. Statistical Analysis

Continuous variables were reported as mean ± standard deviation. Rates or categorical parameters were reported as numbers and percentages. Between-group differences were assessed using the paired t-test for continuous data and correlation with Spearman’s rho. A *p* < 0.05 was considered statistically significant.

Statistical analysis was carried out with GraphPad InStat software (Version 3.06 for Windows, San Diego, CA, USA).

## 3. Results

### 3.1. Population

A total of 466 male partners of infertile couples who were referred to the Andrology Unit and were candidates for genetic tests for a condition of severe oligozoospermia/azoospermia were evaluated.

The test revealed a genetic alteration in 72 patients (15.5%). The mean age ± SD was 33.6 ± 9.1 years. Patients were divided based on the type of anomaly:

- 29 patients had chromosomal abnormalities; among them, 22 patients (30.6%, mean age of 34.2 ± 11.8 years) had 47, XXY karyotype, and 7 patients (9.7%, mean age of 31.9 ± 7.1 years) had other chromosomal abnormalities (Table 1).
- 23 patients (31.9%, mean age of 35.4 ± 6.4 years) carriers of at least one disease-causing pathogenic variant or hypomorphic variant in the *CFTR* gene (Table 2); specifically, in 3 patients, two mutations were found in heterozygosity (F508del plus T338I; D1152H plus D1270N, and F508del; G542X), while in the other 2 patients, the polymorphic IVS8 5T was found in homozygosity and in one patient in association with a mutation.
- 14 patients (19.4%, mean age of 31 ± 8.5 years) with Y chromosome microdeletions (Table 3).
- 6 patients (8.3%, mean age of 32.7 ± 10.7 years) with CHH associated with Kallmann syndrome. Of them, 4/6 (66.7%) received treatment with testosterone for delayed puberty before fertility assessment evaluation in our center. None of them were received before genetic diagnosis.
  No significant differences in age between subgroups were observed.

### 3.2. Semen Parameters

The results of semen parameters are described in Table 4. The prevalence of azoospermia was 80.6% and of oligozoospermia 19.4%.

The prevalence of azoospermia was significantly higher in patients with 47, XXY karyotype (19, 100%) than in the groups with other chromosomal abnormalities (3, 42.9%; *p* < 0.001), *CFTR* gene mutations (17, 73.9%; *p* = 0.02), and Y microdeletion (10, 71.4%; *p* = 0.02).

The prevalence of azoospermia was significantly higher in patients with Kallmann syndrome (6, 100%) than in patients with chromosomal abnormalities (*p* = 0.03).

Considering sperm parameters in non-azoospermic patients, motility was significantly higher in patients with *CFTR* gene mutations than in patients with Y microdeletion (*p* = 0.02), while no differences were observed for sperm morphology abnormalities.

The pH of patients with *CFTR* gene mutations was significantly lower than in patients with Kallmann syndrome, with 47, XXY karyotype, chromosomal abnormalities, and Y microdeletion (*p* < 0.001). Additionally, the volume of patients with *CFTR* gene mutations was significantly lower than those with Kallmann syndrome and Y Microdeletion (*p* < 0.001). The volume of patients with 47, XXY karyotype was significantly lower compared to patients with Y microdeletion and Kallmann syndrome (*p* < 0.001).

### 3.3. Hormonal Profile

The results of the hormonal profile are described in Table 5. Gonadotrophin levels in patients with Kallmann syndrome were significantly lower than in patients with 47, XXY karyotype, Y microdeletion, and *CFTR* gene mutations (*p* < 0.001).

Testosterone value was significantly lower in patients with Kallmann syndrome than in patients with 47, XXY karyotype, Y microdeletion, *CFTR* gene mutations, and chromosomal abnormalities (*p* < 0.05).

Gonadotropin concentrations in patients with 47, XXY karyotype were significantly higher than in patients with *CFTR* gene mutations and chromosomal abnormalities (FSH *p* < 0.001 and LH *p* = 0.006); on the other hand, compared to the group of patients with Y microdeletion, only the hormone FSH was significantly higher (*p* = 0.047). FSH values were significantly higher in patients with Y microdeletion than those with *CFTR* gene mutations (*p* = 0.046). No significant differences were observed in the remaining comparisons.

### 3.4. Testicular and Epididymis Ultrasound

The results of testicular and epididymis ultrasound described in Table 6 were available for 55 patients. The prevalence of testicular hypoplasia was significantly higher in patients with 47, XXY karyotype than in patients with other chromosomal abnormalities (*p* = 0.01), Y microdeletion (*p* = 0.02), and *CFTR* gene mutations (*p* < 0.001). The testicular volume of patients with Y microdeletion was significantly lower than in patients with *CFTR* gene mutations (*p* = 0.004). The prevalence of intratesticular cysts and/or calcifications was significantly higher in patients with 47, XXY karyotype than in patients with *CFTR* gene mutations (*p* = 0.01). The prevalence of testicular inhomogeneity was higher in patients with 47, XXY karyotype, even if not statistically significant.

The incidence of varicocele was significantly higher in patients with Y microdeletion than in patients with 47, XXY karyotype (*p* = 0.003).

No significant difference was found when comparing the prevalence of epididymal abnormalities in different populations. Bilateral or monolateral absence of the vas deferens was observable in all patients with *CFTR* gene mutations (Table 6).

## 4. Discussion

In this study, a total of 466 male partners of infertile couples were evaluated, and a genetic etiology was identified in 15.4% of cases. These results agree with the literature data, which demonstrates that 15% of male infertility cases consist of a genetic disease determined by a specific alteration of a gene or by a chromosomal anomaly of a structural or numerical type [1].

Considering the prevalence of the specific genetic alteration, *CFTR* gene mutations showed a higher prevalence, covering 31.9% of the sample and 4.9% of the entire population. This prevalence appears to be higher than that reported in the literature. Indeed, the percentage of heterozygous mutations associated with *CFTR* gene mutations in the Caucasian population is 5%, while the prevalence of CBVAD in infertile patients is 2% [17,18,19]. The most frequent mutation found in the study population is F508del (31% of patients), a Class II mutation with a worldwide frequency of 70% represents the leading mutation in Northern Europe [15]. The second most frequent mutations observed consist of W1282X (13% of patients), a Class I mutation with a high prevalence in the Ashkenazi race, and N1303K (13% of patients), a Class II mutation with a frequency of 1.3% in Italy [20]. The IVS8-5T polymorphic allele has a high prevalence in the Caucasian population and consists of a Class V mutation, which does not directly determine the disease but can reduce the functionality of the protein; this variant was found in 17.2% of study patients. Probably, the greater frequency in our population is because the diagnosis was also made in patients without CBVAD and, therefore, partial obstruction.

As regards karyotype anomalies, KS represents the first cause of infertility due to genetic causes in the general population, with a prevalence of up to 15% in subjects with NOA, which could yet be underestimated [5,17,21]. In this study, it represents the most frequent karyotype anomaly. Specifically, a 47, XXY karyotype was observed in 86% of cases, while the mosaic form was found in only 3 patients (13.6%).

Structural chromosomal aberrations accounted for 9.7% of genetic anomalies and 1.5% of all infertile patients; Robertsonian translocations were the most frequent chromosomal abnormality, with the observation of the forms rob(13;14)(q10;q10) and rob(14;21)(q10;q10), which are, respectively, the first and the second most commonly observed form in the general population [22]. The other anomalies found consist of two apparently balanced translocations, one between chromosomes 1 and 18 (46 XY, t(1;18)(p22;q21)) and a complex one between chromosomes 4, 11, and 9 (46XY,t(4;11;9)), both characterized by azoospermia. These two translocations are of particular interest because, to our knowledge, they have been associated with azoospermia condition for the first time.

Similarly, 46 XY, r(20) karyotypes have not been described, to date, as a cause of sperm parameter alteration, differently from 13, 21, 22, and Y ring chromosomes [23]. We can speculate that alteration of the seminal parameters could be a consequence of the instability of the ring chromosomes, which during the mitotic processes lead to the delay of anaphase and aneuploidy, thus determining the arrest of the cell cycle and the initiation of [23].

Y chromosome microdeletions are the second most frequent cause of genetic infertility, with an incidence of up to 15% in patients with NOA [7]. In our population, this condition represents 19.4% of the study population. AZFc is the most frequently observed microdeletion in the sample. This is also confirmed by the literature data, where 70% of Y microdeletions involve AZFc.

Considering semen parameters, we found 80.6% of patients with azoospermia; among them, 70.7% had NOA forms, and 29.3% had OA. These data are in agreement with the literature [24]. KS can be associated with both azoospermia and, less frequently, severe oligozoospermia. In this study, however, all patients were azoospermic; probably, our setting is represented by patients with relatively high age and, subsequently, compromised testicular function [25].

Azoospermic patients with heterozygous mutations of *CFTR* had the lowest volume and pH of the entire population, confirming the picture of OA secondary to CBAVD. Conventionally, CBAVD is determined by the presence of a severe mutation associated with a mild mutation or by the presence of two mild mutations; the presence of two severe mutations determines a picture of CF with CBAVD [26]. Two mutations were found in only three patients: one patient with one severe mutation and one mild mutation (F508del; T338I), one patient with two mild mutations (D1152H; D1270N), and one patient with two severe mutations (F508del; G542X). The combination of these two mutations is known to result in multi-system disease; however, the diagnosis was made only following the detection of CBAVD, which represented the only clinical manifestation of the disease in this patient. The ISV8 5T polymorphic allele was observed in homozygosity in two patients, one of which had oligozoospermia and the other azoospermia; on the contrary, when the allele was associated with another mutation, the resulting phenotype was always a picture of CBAVD. The results of this study confirm the complexity of the pathogenic mechanisms of CBAVD, the wide heterogeneity of mutations affecting the *CFTR* gene, and the absence of a unique correlation between a mutation and a clinical phenotype. However, in subjects with only one variant in the *CFTR* gene, it was not possible to investigate possible rarer variants in the other copy of the gene by sequencing all the coding regions of the gene (second-level examination in NGS). Furthermore, in patients with two variants, it was not possible to determine the phase: whether one variant on one copy of *CFTR* and the other on the second, in trans, or both on a single pair, in cis (in the first case confirming a genetic diagnosis, in the second case not confirming it, and the patient would be a simple heterozygote).

Considering patients with Y microdeletion, our data confirm the results described in the literature. In fact, patients with AZFc deletion presented oligozoospermia while in the presence of complete deletions of AZFa and AFZb were azoospermic; these deletions exhibit the distinct clinical phenotypes of Sertoli cell-only syndrome and spermatogenic arrest. Severe oligospermia is confirmed by studies, in which 83% of the population manifests itself with a sperm concentration of 0.1 million/mL [24].

Concerning the hormonal profile, we found high gonadotropin concentrations in subjects with 47, XXY karyotype and low in subjects with Kallmann syndrome, as expected. However, the FSH concentration in our KS patients was higher than in patients from the Italian population (29.5 ± 12.5 vs. 22.6 ± 13.8 mlU/mL, *p* = 0.03). This difference is likely related to the higher age in the present study compared to the other group (34.2 ± 11.8 vs. 27.5 ± 9.6 years; *p* = 0.004) [22]. Patients with Y microdeletions were characterized by normal testosterone and LH levels and by an elevated FSH concentration, an expression of widespread testicular damage. Comparing the gonadotropin concentration with the values reported by other studies conducted in Italy, no significant difference was found [24,27]. On the other hand, patients with CF present a normal hormonal balance from a clinical point of view since there is no testicular damage, as expected.

Finally, considering ultrasound results, 63% of the study population is characterized by testicular atrophy, mainly in KS and Kallmann syndromes. Interestingly, anomalies of the testicular homogeneity were found to be higher in the subgroups with KS and Y microdeletions. This observation is to be expected, given that CBAVD is a form of post-testicular azoospermia without intrinsic damage to the gonad. The ultrasound characteristics in patients with KS reflect the data present in the literature, where on ultrasound examination, the testicles appear small with a markedly inhomogeneous echostructure or with microlithiasis [28]. Furthermore, the lesions in these patients are often tumors or benign Leydig cell hyperplasia [28].

Indeed, one patient with KS in our series had previously undergone an orchidectomy for the presence of a Leydig cell tumor [29].

The incidence of testicular microlithiasis in the infertile population in the literature ranges from 0.8% to 15%, reaching 33% in genetic disorders [29]; in our study, it was detected in 12.8%, mainly found in patients with KS and Y microdeletion. The incidence of varicocele in the study population was high in patients with Y Microdeletion, reaching 91.6%. However, no correlation has been found between these two forms of infertility; in fact, several studies have searched for a possible association without success, looking for the genetic abnormality in infertile patients with varicocele [30].

## 5. Conclusions

In conclusion, the prevalence of genetic anomalies in a population of male partners of infertile couples was 15.4%, in line with the literature data. Mutations linked to the *CFTR* gene were more frequent, with clinical features ranging from azoospermia to oligozoospermia, highlighting the complexity and heterogeneity of presentation. Furthermore, the alterations given by the ring chromosomes in gametogenesis, as well as other translocation, have not been described and, therefore, further investigations are needed to understand if the condition of oligozoospermia in these patients is an incidental factor or if it is related to the genetic anomaly. This study emphasizes the importance of accurate genetic screening in infertile patients for the purpose of correctly identifying the causal factor as well as appropriate treatment.

## Figures and Tables

**Table 1:** Karyotype profile and related sperm alterations. OAT = oligoasthenoteratozoospermia.

Number of Patients	Karyotype	Sperm Characteristic
19 (65.5%)	47, XXY	Azoospermia
3 (10.3%)	46, XY/47, XXY	OAT (2)/Azoospermia (1)
1 (3.4%)	XX, SRY+	Azoospermia
1 (3.4%)	45, XY, der(14;21)(q10;q10)	OAT
1 (3.4%)	45, XY, rob(13;14)(q10;q10)	OAT
1 (3.4%)	46, XY, t(4;11;9)	Azoospermia
1 (3.4%)	46, XY, t(1;18)(p22;q21)	Azoospermia
1 (3.4%)	46, XY, r(20)	Oligozoospermia
1 (3.4%)	46, X, inv(Y)	Oligozoospermia

**Table 2:** Cystic fibrosis gene variants. OAT = oligoasthenoteratozoospermia.

N° of Patients per Mutation	Nucleotide Change	Aminoacid Change	Traditional Nomenclature	dbSNP	Sperm Characteristic
9 (31%)	c.1521_1523delCTT	p.(Phe508del)	F508del	rs113993960	Azoospermia (8)/OAT (1)
5 (17.2%)	c. 1210-12T [5]	p.?	IVS8-5T	rs1805177	Azoospermia (4)/oligoasthenozoospermia (1)
3 (10.3%)	c.3846G>A	p.(Trp1282X)	W1282X	rs77010898	Azoospermia
3 (10.3%)	c.3909C>G	p.(Asn1303Lys)	N1303K	rs80034486	Oligoasthenozospermia
2 (6.9%)	c.1624G>T	p.(Gly542Ter)	G542X	rs113993959	Azoospermia
1 (3.4%)	c.1040G>C	p.(Arg347Pro)	R347P	rs77932196	Azoospermia
1 (3.4%)	c.3454G>C	p.(Asp1152His)	D1152H	rs75541969	Azoospermia
1 (3.4%)	c.350G>A	p.(Arg117His)	R117H	rs78655421	Azoospermia
1 (3.4%)	c.4046G>A	p.(Gly1349Asp)	G1349D	rs193922525	Azoospermia
1 (3.4%)	c.2051_2052delinsG	p.(Lys684fs)	2184AA>G	rs121908799	Azoospermia
1 (3.4%)	c.1013C>T	p.(Thr338Ile)	T338I	rs77409459	Azoospermia
1 (3.4%)	c.3808G>A	p.Asp1270Asn	D1270N	rs11971167	Azoospermia

**Table 3:** Y chromosome microdeletion. OAT = oligoasthenoteratozoospermia.

Number of Patients	Type of Microdeletion	Sperm Characteristic
8 (57.1%)	AZFc	OAT (4)/Azoospermia (4)
2 (14.3%)	AZFbc	Azoospermia
1 (7.1%)	locus DYF51S1	Azoospermia
1 (7.1%)	locus DAZ and DYS240	Azoospermia
1 (7.1%)	AZFa	Azoospermia
1 (7.1%)	AZFb	Azoospermia

**Table 4:** Semen parameters.

	Azoospermia (80.6%)		Oligozoospermia (19.4%)				
	Volume (mL)	pH	Volume (mL)	pH	Concentration (n. ×10^6^)	Total Motility (%)	Abnormal Morphology (%)
Total	2 ± 1.4	7.3 ± 0.5	2.8 ± 0.1	7.4± 0.1	3.5 ± 3.8	9.7 ± 9.4	96 ± 3.5
Karyotype abnormalities	3.4 ± 0.7	7.7 ± 0.3	2.5 ± 1.2	7.4 ± 0.1	4.3 ± 2.9	9.5 ± 11	95 ± 3.2
Y microdeletion	1.3 ± 1.1 _A_	7.7 ± 0.3	2.1 ± 0.8	7.4 ± 0.2	0.4 ± 0.4	0	100
Cystic fybrosis	2.4 ± 2.8	7.5 ± 0.1	2.7 ± 1.5	7.5 ± 0.1	4.7 ± 4.4	10.1 ± 7.2 _D_	94.7± 4.9
Kallmann	3.7 ± 1.1	7.5 ± 0.2	-	-	-	-	-
47, XXY karyotype	1.2 ± 0.9 _B_	6.8 ± 0.7 _C_	-	-	-	-	-

_A_ *p* < 0.05 vs. Kallmann syndrome and Y microdeletion. _B_ *p* < 0.05 vs. Kallmann syndrome and Y microdeletion. _C_ *p* < 0.05 vs. all groups. _D_ *p* < 0.05 vs. Y microdeletion.

**Table 5:** Hormonal profile.

	Testosterone (ng/mL)	FSH (mlU/mL) (1.5–12.4)	LH (mlU/mL) (1.8–12.0)
	(2.6–10.8)		
Total	4.3 ± 2.3	14.2 ± 13.5	7.8 ± 6.6
Kallmann	2.1 ± 1.1 _A_	2 ± 1.7 _B_	0.1 ± 0.1 _B_
47, XXY karyotype	3.4 ± 1.6	29.5 ± 12.5 _C_	14.9 ± 6.4 _D_
Karyotype abnormalities	4.7 ± 1.4	8.6 ± 6.7	5.1 ± 4.8
Y microdeletion	4.2 ± 1.3	17.0 ± 11.6 _E_	9.2 ± 6.5
Cystic fibrosis	5.5 ± 2.9	5.6 ± 4.7	4.7 ± 1.9

_A_ *p* < 0.05 vs. Klinefelter syndrome, karyotype abnormalities, Y microdeletion, and cystic fibrosis. _B_ *p* < 0.05 vs. Klinefelter syndrome, Y microdeletion, and cystic fibrosis. _C_ *p* < 0.05 vs. karyotype abnormalities, Y microdeletion, and cystic fibrosis. _D_ *p* < 0.05 vs. karyotype abnormalities and cystic fibrosis. _E_ *p* < 0.05 vs. cystic fibrosis.

**Table 6:** Testicular and epididymis ultrasound.

	Testicular Hypoplasia (Volume < 12 mL)	Testicular Echostructure/Echogenicity Anomalies		
		Testicular Inhomogeneity	Calcifications/Microcalcifications	Cysts
Total (55/72)	63%	3.7%	12.8%	12.8%
Kallmann (3/6)	100%	/	33.3%	/
47, XXY karyotype (17/22)	100% _A_	33.3%	23.5% _C_	23.5% _C_
Karyotype abnormalities (6/7)	50%	/	/	16.7%
Y microdeletion (12/14)	66.7% _B_	8.3%	25%	16.7%
Cystic fibrosis (17/23)	12.5%	/	5.9% _D_	/
	Enlarged Epididymis/Hyperechogenicity	Cysts	Varicocele I–II	Varicocele III
Total (55/72)	43.6%	29.1%	56.3%	3.6%
Kallmann (3/6)	33.3%	33.3%	66.7%	/
47, XXY karyotype (17/22)	35.3%	23.5%	35.3%	/
Karyotype abnormalities (6/7)	33.3%	16.7%	66.7%	/
Y microdeletion (12/14)	58.3%	41.7%	83.3% _A_	8.3%
Cystic fibrosis (17/23)	47.1%	29.4%	52.9%	5.8%

_A_ *p* < 0.05 vs. karyotype abnormalities, Y microdeletion, and cystic fibrosis. _B_ *p* < 0.05 vs. cystic fibrosis. _C_ *p* < 0.05 vs. cystic fibrosis. _D_ *p* < 0.05 vs. Kallmann.

## Data Availability

The raw data supporting the conclusions of this article will be made available by the authors on request.
